# Supplementary material for: On the joint role of non-Hispanic Black race/ethnicity and weight status in predicting postmenopausal weight gain
Source: PLoS One. 2021 Mar 1;16(3):e0247821. doi: 10.1371/journal.pone.0247821 (PMC7920337; doi:10.1371/journal.pone.0247821)
Supplement: S1 Table — (DOCX) [file pone.0247821.s002.docx]

| S1 Table. Common referent hazard ratios and 95% confidence intervals comparing the hazard of a ≥10% weight gain by baseline weight status in non-Hispanic Blacks and non-Hispanic Whites stratified by age (n=70,750) ^1-3^ | | | | | | | |
| --- | --- | --- | --- | --- | --- | --- | --- |
|  |  | Hazard ratios (95% confidence interval) | | | | |  |
|  |  | Normal weight | Overweight | Obese class I | Obese class II | Obese class III | *p*-trend |
| Ages 49 - 54 | Crude models |  |  |  |  |  |  |
|  | Non-Hispanic White | 1.00 (ref.) | 1.17 (1.09, 1.26) | 1.17 (1.05, 1.29) | 1.18 (1.02, 1.37) | 1.03 (0.84, 1.25) | 0.003 |
|  | Non-Hispanic Black^4^ | 1.89 (1.57, 2.27) | 1.57 (1.34, 1.85) | 1.35 (1.08, 1.68) | 1.64 (1.19, 2.25) | 1.50 (1.05, 2.14) | 0.115 |
|  | Adjusted models |  |  |  |  |  |  |
|  | Non-Hispanic White | 1.00 (ref.) | 1.12 (1.04, 1.20) | 1.06 (0.96, 1.18) | 1.04 (0.89, 1.20) | 0.89 (0.73, 1.08) | 0.829 |
|  | Non-Hispanic Black^4^ | 1.59 (1.32, 1.91) | 1.36 (1.15, 1.61) | 1.10 (0.88, 1.38) | 1.27 (0.92, 1.76) | 1.17 (0.82, 1.67) | 0.203 |
| Ages 55 - 59 | Crude models |  |  |  |  |  |  |
|  | Non-Hispanic White | 1.00 (ref.) | 1.13 (1.05, 1.22) | 1.26 (1.15, 1.39) | 1.16 (1.00, 1.35) | 0.89 (0.72, 1.09) | 0.005 |
|  | Non-Hispanic Black^4^ | 1.80 (1.47, 2.20) | 1.34 (1.11, 1.61) | 1.57 (1.26, 1.95) | 1.44 (1.04, 2.00) | 1.03 (0.69, 1.53) | 0.040 |
|  | Adjusted models |  |  |  |  |  |  |
|  | Non-Hispanic White | 1.00 (ref.) | 1.15 (1.03, 1.28) | 1.27 (1.10, 1.46) | 1.00 (0.80, 1.26) | 0.69 (0.49, 0.96) | 0.657 |
|  | Non-Hispanic Black^4^ | 1.63 (1.22, 2.19) | 1.10 (0.82, 1.49) | 1.09 (0.73, 1.64) | 0.83 (0.42, 1.65) | 1.07 (0.56, 2.04) | 0.218 |
| Ages 60 - 64 | Crude models |  |  |  |  |  |  |
|  | Non-Hispanic White | 1.00 (ref.) | 0.96 (0.92, 1.01) | 0.93 (0.87, 1.00) | 1.09 (0.98, 1.22) | 0.93 (0.80, 1.09) | 0.435 |
|  | Non-Hispanic Black^4^ | 1.79 (1.53, 2.08) | 1.48 (1.30, 1.68) | 1.33 (1.13, 1.56) | 1.70 (1.34, 2.17) | 1.60 (1.19, 2.15) | 0.279 |
|  | Adjusted models |  |  |  |  |  |  |
|  | Non-Hispanic White | 1.00 (ref.) | 0.97 (0.90, 1.05) | 0.80 (0.71, 0.91) | 0.84 (0.70, 1.02) | 0.62 (0.46, 0.82) | <0.001 |
|  | Non-Hispanic Black^4^ | 1.30 (0.97, 1.75) | 1.02 (0.78, 1.32) | 1.09 (0.80, 1.49) | 0.86 (0.47, 1.58) | 0.69 (0.31, 1.52) | 0.262 |
| 65 and older | Crude models |  |  |  |  |  |  |
|  | Non-Hispanic White | 1.00 (ref.) | 0.94 (0.88, 1.00) | 0.88 (0.80, 0.96) | 1.12 (0.97, 1.29) | 0.85 (0.67, 1.09) | 0.118 |
|  | Non-Hispanic Black^4^ | 1.80 (1.48, 2.19) | 1.53 (1.29, 1.82) | 1.43 (1.15, 1.79) | 1.49 (1.01, 2.20) | 1.77 (1.16, 2.69) | 0.373 |
|  | Adjusted models |  |  |  |  |  |  |
|  | Non-Hispanic White | 1.00 (ref.) | 0.91 (0.85, 0.97) | 0.81 (0.74, 0.89) | 0.98 (0.85, 1.14) | 0.72 (0.56, 0.92) | <0.001 |
|  | Non-Hispanic Black^4^ | 1.48 (1.22, 1.81) | 1.25 (1.05, 1.48) | 1.12 (0.90, 1.40) | 1.17 (0.79, 1.74) | 1.29 (0.84, 1.96) | 0.154 |
| Abbreviations: BMI, body mass index; MET, metabolic equivalent; ref., referent group | | | | | | | |
| ^1^ Weight status was defined using baseline body mass index (BMI) as normal weight (BMI: 18.5-24.9 kg/m^2^), overweight (BMI: 25.0-29.9 kg/m^2^), obese class I (BMI: 30.0-34.9 kg/m^2^), obese class II (BMI: 35.0-39.9 kg/m^2^), or obese class III (BMI ≥40.0 kg/m^2^) | | | | | | | |
| ^2^ Adjusted models controlled for education level, annual household income, smoking status, alcohol intake, age, total energy intake at baseline and MET-hours of mild, moderate and hard exercise | | | | | | | |
| ^3^ *P*-trend corresponds to a Wald test statistic when a linear term for baseline body weight status was substituted in the model | | | | | | | |
| ^4^ Values shown are relative to the common referent group, normal weight non-Hispanic Whites | | | | | | | |
