# Supplementary material for: On the joint role of non-Hispanic Black race/ethnicity and weight status in predicting postmenopausal weight gain
Source: PLoS One. 2021 Mar 1;16(3):e0247821. doi: 10.1371/journal.pone.0247821 (PMC7920337; doi:10.1371/journal.pone.0247821)
Supplement: S2 Table — (DOCX) [file pone.0247821.s003.docx]

| S2 Table. Overall and stratum-specific hazard ratios and 95% confidence intervals comparing the hazard for ≥10% weight gain by baseline weight status in non-Hispanic Blacks and non-Hispanics stratified by age (n=70,750)^1-3^ | | | | | | | |
| --- | --- | --- | --- | --- | --- | --- | --- |
|  |  | Hazard ratios (95% confidence interval) | | | | |  |
|  |  | Normal weight | Overweight | Obese class I | Obese class II | Obese class III | *p*-trend |
| Ages 49 - 54 | Crude models |  |  |  |  |  |  |
|  | Overall | 1.00 (ref.) | 1.17 (1.08, 1.27) | 1.16 (1.03, 1.29) | 1.22 (1.04, 1.42) | 1.08 (0.89, 1.30) | <0.001 |
|  | Non-Hispanic Black | 1.00 (ref.) | 0.84 (0.66, 1.05) | 0.71 (0.54, 0.93) | 0.85 (0.60, 1.21) | 0.77 (0.53, 1.13) | 0.115 |
|  | Adjusted models |  |  |  |  |  |  |
|  | Overall | 1.00 (ref.) | 1.11 (1.02, 1.21) | 1.04 (0.93, 1.16) | 1.04 (0.89, 1.22) | 0.91 (0.75, 1.10) | 0.961 |
|  | Non-Hispanic Black | 1.00 (ref.) | 0.84 (0.66, 1.06) | 0.74 (0.56, 0.97) | 0.88 (0.62, 1.26) | 0.80 (0.54, 1.18) | 0.203 |
| Ages 55 - 59 | Crude models |  |  |  |  |  |  |
|  | Overall | 1.00 (ref.) | 1.12 (1.04, 1.20) | 1.26 (1.15, 1.38) | 1.17 (1.02, 1.34) | 0.89 (0.74, 1.07) | 0.005 |
|  | Non-Hispanic Black | 1.00 (ref.) | 0.75 (0.58, 0.97) | 0.86 (0.65, 1.14) | 0.79 (0.55, 1.14) | 0.57 (0.37, 0.88) | 0.040 |
|  | Adjusted models |  |  |  |  |  |  |
|  | Overall | 1.00 (ref.) | 1.12 (1.01, 1.24) | 1.23 (1.07, 1.41) | 0.96 (0.77, 1.20) | 0.72 (0.53, 0.97) | 0.997 |
|  | Non-Hispanic Black | 1.00 (ref.) | 0.63 (0.41, 0.97) | 0.68 (0.41, 1.14) | 0.54 (0.25, 1.17) | 0.76 (0.35, 1.63) | 0.218 |
| Ages 60 - 64 | Crude models |  |  |  |  |  |  |
|  | Overall | 1.00 (ref.) | 0.97 (0.93, 1.02) | 0.95 (0.89, 1.01) | 1.13 (1.03, 1.25) | 1.00 (0.88, 1.15) | 0.709 |
|  | Non-Hispanic Black | 1.00 (ref.) | 0.83 (0.68, 1.00) | 0.74 (0.60, 0.91) | 0.94 (0.71, 1.23) | 0.88 (0.64, 1.21) | 0.279 |
|  | Adjusted models |  |  |  |  |  |  |
|  | Overall | 1.00 (ref.) | 0.97 (0.89, 1.05) | 0.82 (0.73, 0.92) | 0.83 (0.70, 1.00) | 0.62 (0.47, 0.81) | <0.001 |
|  | Non-Hispanic Black | 1.00 (ref.) | 0.82 (0.55, 1.24) | 0.84 (0.53, 1.34) | 0.74 (0.36, 1.50) | 0.64 (0.26, 1.58) | 0.262 |
| 65 and older | Crude models |  |  |  |  |  |  |
|  | Overall | 1.00 (ref.) | 0.95 (0.89, 1.02) | 0.90 (0.82, 0.99) | 1.13 (0.98, 1.30) | 0.95 (0.77, 1.18) | 0.463 |
|  | Non-Hispanic Black | 1.00 (ref.) | 0.85 (0.66, 1.09) | 0.79 (0.59, 1.05) | 0.81 (0.53, 1.24) | 0.96 (0.61, 1.51) | 0.373 |
|  | Adjusted models |  |  |  |  |  |  |
|  | Overall | 1.00 (ref.) | 0.91 (0.85, 0.98) | 0.82 (0.75, 0.90) | 0.98 (0.85, 1.13) | 0.78 (0.63, 0.97) | <0.001 |
|  | Non-Hispanic Black | 1.00 (ref.) | 0.84 (0.65, 1.09) | 0.74 (0.55, 1.00) | 0.79 (0.51, 1.22) | 0.83 (0.53, 1.31) | 0.154 |
| Abbreviations: BMI, body mass index; MET, metabolic equivalent; ref., referent group | | | | | | | |
| ^1^ Weight status was defined using baseline body mass index (BMI) as normal weight (BMI: 18.5-24.9 kg/m^2^), overweight (BMI: 25.0-29.9 kg/m^2^), obese class I (BMI: 30.0-34.9 kg/m^2^), obese class II (BMI: 35.0-39.9 kg/m^2^), or obese class III (BMI ≥40.0 kg/m^2^) | | | | | | | |
| ^2^ Adjusted models controlled for education level, annual household income, smoking status, alcohol intake, age, total energy intake at baseline and MET-hours of mild, moderate and hard exercise | | | | | | | |
| ^3^ *P*-trend corresponds to a Wald test statistic when a linear term for baseline body weight status was substituted in the model | | | | | | | |
