# Supplementary material for: On the joint role of non-Hispanic Black race/ethnicity and weight status in predicting postmenopausal weight gain
Source: PLoS One. 2021 Mar 1;16(3):e0247821. doi: 10.1371/journal.pone.0247821 (PMC7920337; doi:10.1371/journal.pone.0247821)
Supplement: S3 Table — (DOCX) [file pone.0247821.s004.docx]

| S3 Table. Overall and common referent hazard ratios and 95% confidence intervals comparing the hazard for ≥10% weight gain by baseline weight status in non-Hispanic Blacks and non-Hispanics using complete-case analysis ^1-3^ | | | | | | |
| --- | --- | --- | --- | --- | --- | --- |
|  | Common referent (normal weight, non-Hispanic Whites) hazard ratios (95% confidence interval) | | | | |  |
|  | Normal weight | Overweight | Obese class I | Obese class II | Obese class III | *p*-trend |
| Crude models |  |  |  |  |  |  |
| Overall | 1.00 (ref.) | 1.03 (0.99, 1.07) | 1.05 (1.00, 1.11) | 1.17 (1.09, 1.26) | 1.00 (0.91, 1.11) | <0.001 |
| Non-Hispanic White | 1.00 (ref.) | 1.02 (0.99, 1.06) | 1.03 (0.98, 1.09) | 1.13 (1.05, 1.22) | 0.95 (0.85, 1.06) | 0.066 |
| Non-Hispanic Black | 1.78 (1.61, 1.97) | 1.49 (1.37, 1.63) | 1.46 (1.31, 1.63) | 1.72 (1.45, 2.03) | 1.43 (1.17, 1.74) | 0.052 |
| Non-Hispanic Black | 1.0 (ref.) | 0.84 (0.74, 0.95) | 0.81 (0.70, 0.93) | 0.94 (0.78, 1.13) | 0.78 (0.63, 0.97) | 0.052 |
| Adjusted models |  |  |  |  |  |  |
| Overall | 1.00 (ref.) | 1.00 (0.96, 1.04) | 0.96 (0.91, 1.01) | 0.98 (0.91, 1.06) | 0.80 (0.72, 0.89) | <0.001 |
| Non-Hispanic White | 1.00 (ref.) | 1.00 (0.97, 1.04) | 0.96 (0.92, 1.01) | 0.97 (0.89, 1.05) | 0.78 (0.70, 0.87) | <0.001 |
| Non-Hispanic Black | 1.42 (1.28, 1.57) | 1.20 (1.09, 1.32) | 1.09 (0.96, 1.22) | 1.24 (1.04, 1.49) | 1.01 (0.81, 1.25) | 0.007 |
| Non-Hispanic Black | 1.0 (ref.) | 0.84 (0.73, 0.96) | 0.77 (0.66, 0.89) | 0.89 (0.73, 1.09) | 0.73 (0.58, 0.92) | 0.007 |
| Abbreviations: BMI, body mass index; ref., referent group | | | | | | |
| ^*^ Hazard ratio was significantly different from the referent value, *p*<0.05 | | | | | | |
| ^ⱡ^ Stratum-specific hazard ratio in non-Hispanic Blacks was statistically different from that of non-Hispanic Whites, *p*<0.05 | | | | | | |
| ^1^ Weight status was defined using baseline body mass index (BMI) as normal weight (BMI: 18.5-24.9 kg/m^2^), overweight (BMI: 25.0-29.9 kg/m^2^), obese class I (BMI: 30.0-34.9 kg/m^2^), obese class II (BMI: 35.0-39.9 kg/m^2^), or obese class III (BMI ≥40.0 kg/m^2^) | | | | | | |
| ^2^ Adjusted models controlled for education level, annual household income, smoking status, alcohol intake, age and MET-hours of mild, moderate and hard exercise. | | | | | | |
| ^3^ *P*-trend corresponds to a Wald test statistic when a linear term for baseline body weight status was substituted in the model | | | | | | |
| ^4^ In crude models within-strata differences were not statistically different by race/ethnicity (α = 0.05) | | | | | | |
| ^5^ In adjusted models within-strata differences were not statistically different by race/ethnicity (α = 0.05) | | | | | | |
