# Supplementary material for: On the joint role of non-Hispanic Black race/ethnicity and weight status in predicting postmenopausal weight gain
Source: PLoS One. 2021 Mar 1;16(3):e0247821. doi: 10.1371/journal.pone.0247821 (PMC7920337; doi:10.1371/journal.pone.0247821)
Supplement: S4 Table — (DOCX) [file pone.0247821.s005.docx]

| S4 Table. Overall and common referent hazard ratios and 95% confidence intervals comparing the hazard for ≥10 pound weight gain by baseline weight status in non-Hispanic Blacks and non-Hispanics ^1-3^ | | | | | | |
| --- | --- | --- | --- | --- | --- | --- |
|  | Common referent (normal weight, non-Hispanic Whites) hazard ratios (95% confidence interval) | | | | |  |
|  | Normal weight | Overweight | Obese class I | Obese class II | Obese class III | *p*-trend |
| Crude models |  |  |  |  |  |  |
| Overall | 1.00 (ref.) | 1.00 (0.88, 1.14) | 1.20 (1.02, 1.41) | 1.19 (0.92, 1.54) | 1.37 (1.01, 1.86) | 0.005 |
| Non-Hispanic White | 1.00 (ref.) | 1.00 (0.88, 1.15) | 1.19 (1.00, 1.41) | 1.10 (0.83, 1.46) | 1.36 (0.98, 1.90) | 0.026 |
| Non-Hispanic Black | 1.74 (1.19, 2.53) | 1.29 (0.91, 1.82) | 1.59 (1.08, 2.36) | 2.15 (1.26, 3.67) | 1.62 (0.82, 3.20) | 0.626 |
| Non-Hispanic Black | 1.0 (ref.) | 0.74 (0.45, 1.22) | 0.92 (0.54, 1.56) | 1.24 (0.65, 2.36) | 0.93 (0.43, 2.01) | 0.626 |
| Adjusted models |  |  |  |  |  |  |
| Overall | 1.00 (ref.) | 0.98 (0.86, 1.12) | 1.16 (0.98, 1.37) | 1.14 (0.88, 1.49) | 1.32 (0.97, 1.80) | 0.029 |
| Non-Hispanic White | 1.00 (ref.) | 0.99 (0.86, 1.13) | 1.16 (0.97, 1.38) | 1.07 (0.80, 1.42) | 1.34 (0.96, 1.87) | 0.064 |
| Non-Hispanic Black | 1.67 (1.14, 2.45) | 1.25 (0.88, 1.77) | 1.52 (1.02, 2.26) | 2.03 (1.18, 3.48) | 1.54 (0.77, 3.07) | 0.757 |
| Non-Hispanic Black | 1.0 (ref.) | 0.72 (0.44, 1.17) | 0.86 (0.51, 1.46) | 1.16 (0.59, 2.27) | 0.92 (0.41, 2.04) | 0.757 |
| Abbreviations: BMI, body mass index; ref., referent group | | | | | | |
| ^*^ Hazard ratio was significantly different from the referent value, *p*<0.05 | | | | | | |
| ^ⱡ^ Stratum-specific hazard ratio in non-Hispanic Blacks was statistically different from that of non-Hispanic Whites, *p*<0.05 | | | | | | |
| ^1^ Weight status was defined using baseline body mass index (BMI) as normal weight (BMI: 18.5-24.9 kg/m^2^), overweight (BMI: 25.0-29.9 kg/m^2^), obese class I (BMI: 30.0-34.9 kg/m^2^), obese class II (BMI: 35.0-39.9 kg/m^2^), or obese class III (BMI ≥40.0 kg/m^2^) | | | | | | |
| ^2^ Adjusted models controlled for education level, annual household income, smoking status, alcohol intake, age and MET-hours of mild, moderate and hard exercise. | | | | | | |
| ^3^ *P*-trend corresponds to a Wald test statistic when a linear term for baseline body weight status was substituted in the model | | | | | | |
| ^4^ In crude models within-strata differences were not statistically different by race/ethnicity (α = 0.05) | | | | | | |
| ^5^ In adjusted models within-strata differences were not statistically different by race/ethnicity (α = 0.05) | | | | | | |
