# Supplementary material for: On the joint role of non-Hispanic Black race/ethnicity and weight status in predicting postmenopausal weight gain
Source: PLoS One. 2021 Mar 1;16(3):e0247821. doi: 10.1371/journal.pone.0247821 (PMC7920337; doi:10.1371/journal.pone.0247821)
Supplement: Attachment — Submitted filename: Response to Reviewers.docx [file pone.0247821.s006.docx]

**Comments from reviewer #1**

**Summary comments:** This manuscript describes weight gain among post-menopausal women in the Women’s Health Initiative, comparing weight gain in non-Hispanic white women and non-Hispanic black women by weight levels at baseline. An important contribution of the study is its ability to look at weight gain comparing women who were normal weight at the beginning of follow-up to women who were overweight or obese due to the large sample size of the cohort. Overall the paper clearly describes the analyses and results and the discussion is generally appropriate.

One of this reviewer’s major concerns was that the mean age of the cohort at baseline was approximately 63 years, meaning that the women were on average 12 years beyond the median age of menopause. When thinking about “post-menopausal weight gain”, for many women the concern is about weight gain in the more immediate post-menopausal period. Given the large size of the cohort, it would be possible and very informative to do analyses stratified by age at baseline (e.g., 50-54, 55-59, 60+). Such analyses would provide very useful information about whether there was a greater risk of weight gain shortly after menopause that leveled off at older ages.

**Response to summary comments**: Thank you for your careful review of this manuscript. Per your suggestion, we have carried out the analyses of Table 2 stratified by age group (using the categories you recommended). We present these findings in Appendix Table 1 and remark on them at lines 209-212 of the revised manuscript. In addition, as noted in our response to **Comment 9** below, we have addressed your concern regarding the age of the sample at baseline at lines 287-291 of the revised manuscript.

Other comments are:

**Comment 1:** Page 3, lines 75-76: The categories of alcohol intake do not seem correct. There may be a missing category between 1-2 drinks per month and 3-6 drinks per week (~12-24 drinks per month).

**Response to comment 1:** Thank you for catching this error. The correct categories for drinking are now given at lines 83-85 of the revised manuscript:

Possible responses ranged from ‘non-drinker’ to `7 or more drinks per week’ (non-drinker, past drinker, <1 drink per month, <1 drink per week, 1-6 drinks per week, or 7 or more drinks per week).

**Comment 2:** Page 3, line 86: Since underweight individuals were excluded, the category <18.5 is not relevant for the women included in this analysis.

**Response to comment 2:** Per your suggestion, we have removed the underweight category at line 95 of the revised manuscript.

**Comment 3:** Page 4, line 94: Should be Cox proportional hazards (not proportion).

**Response to comment 3:** Thank you. We have made the correction at line 103 of the revised manuscript.

**Comment 4:** Page 5, line 113: Should be greater influence (not great).

**Response to comment 4:** Thank you. We have made the correction at line 124 of the revised manuscript.

**Comment 5:** Page 6, line 156: HR 1.70 does not match up with the figure in the table (1.77).

**Response to comment 5:** Thank you for catching this typographical error. We have made the necessary correction at line 168 of the revised manuscript.

**Comment 6:** Table 2 results: The results as presented have overall hazards ratios comparing different overweight/obese categories to normal weight women. For comparison purposes, it would be useful to present parallel results for the Black women – that is, present the HRs comparing overweight and obese black women to normal weight Black women. This would make for a more straightforward comparison than the results presented with normal weight White women as the reference group. (These results could be presented in addition to what is already in the table.)

**Response to comment 6:** Thank you for this helpful suggestion. We have revised Table 2 accordingly, which now includes two additional rows showing the within-strata hazard ratios for non-Hispanic Blacks and non-Hispanic Whites.

**Comment 7:** Also in Table 2, confirm that the p-value for the trend in non-Hispanic White women is accurate. There is basically no indication of a trend in the HRs, but the p-value is close to statistical significance.

**Response to comment 7:** Thank you for raising this concern. We have re-run the analyses and can confirm the p-value for trend now shown in Table 2 is accurate.

**Comment 8:** Page 7, lines 177-179: The statement that “…racial/ethnic divergence in the prevalence of obesity in NHBs and NHWs may begin well in advance of menopause.” While this statement may be true, it is not supported by the data in this study since women were not enrolled in the study until they were on average 12 years past the median age at menopause.

**Response to comment 8:** Thank you for the opportunity to clarify. We have revised the text at lines 223-224, which now states “…racial/ethnic divergence in the prevalence of obesity in NHBs and NHWs may have begun prior to study enrollment.”

**Comment 9:** Page 9, limitations: An additional limitation is that the age of enrollment of the cohort precludes them from making conclusions about weight gain shortly after women go through menopause.

**Response to comment 9:** You are correct to note this as a limitation. Thus, we have added the following text at lines 287-291 of the revised manuscript:

“Finally, it should be noted that the average age of enrollment into the WHI OS was 63.7 years, whereas it has been previously reported that the median age of menopause among US women is 52.6 years (1). Accordingly, for some women in this study, the period immediately after the onset of menopause may not have been captured, which limits our ability to draw conclusions about weight gain during the period shortly after the onset of menopause.”

**Comments from reviewer #2**

**Summary comments:** Thank you for submitting this interesting piece. I think it is a good analysis that comes from a relevant research question. However, I believe the manuscript could be improved in several ways as follows:

**Response to summary comments:** Thank you for your careful review of our manuscript and for your suggestions on how to improve our work.

**Comment 1:** Intro and discussion: in my opinion, there is a rather poor explanation of potential causal mechanisms for the link between menopause, race and weight gain. This is crucial to support the hypothesis and analysis and needs further development. For example, Intro mentions biological, social and environmental factors, but these are not explained.

**Response to comment 1:** Thank you for raising this important concern. We have provided additional text to clarify the social and environmental factors that may contribute to the disparity in postmenopausal weight gain in non-Hispanic Blacks and non-Hispanic Whites. The revised text at lines 50-57 now reads:

“Biological differences in resting energy expenditure and fatty acid metabolism in NHBs and NHWs have been noted and may lead to greater rates of obesity in NHBs compared with NHWs (2-4). Social factors that may contribute to racial disparity in obesity include education level (5), annual household income (6), and physical activity level (7), which are inversely related to risk of weight gain, and tend to be lower in NHBs compared with NHWs (8-10). Environmental factors include limited access to healthy foods (11), and low neighborhood walkability (12), both of which tend to be more common among NHBs than NHWs. Independent of these factors, the extent to which weight status and race/ethnicity interact to influence the risk of postmenopausal weight gain in NHBs versus NHWs is unclear.”

**Comment 2:** Sample selection: underweight women were excluded, this should be justified better, at least for the general description.

**Response to comment 2:** Thank you for the opportunity to clarify our decision to exclude women who were underweight at baseline. At lines 72-74 of the revised manuscript, we made the following addition:-

“We excluded those with underweight BMI (<18,5 kg/m2) at baseline in light of the potential for confounding of the relationship between race/ethnicity and weight gain by chronic diseases associated with wasting (13).”

**Comment 3:** Approach mentions underweight category but this was excluded from analysis, please clarify.

**Response to comment 3:** Thank you. We have removed the description of the underweight category from the methods section, as we agree, it is not relevant in light of these individuals being excluded from the analyses.

**Comment 4:** The main outcome measure at follow up for gain weight is subjective, this needs better consideration in the discussion section. The same variable was measured at different time points, which time point was used for the analysis presented?

**Response to comment 4**: Thank you for the opportunity to clarify. Our main outcome was modeled as a time-to-event outcome, with the event being a 10% weight gain from baseline. Thus, at each of the follow-ups at years 1, 3, 4, 5, 6, 7 and 8, the individual had the potential to develop the outcome, whereupon she would be censored. To better reflect this in our methods, we revised the text at lines 103-106, which now reads:

“Cox proportional hazards models were used to estimate the relationship between race/ethnicity and a ≥10.0% weight gain from baseline. Respondents’ self-reported highest weight since last follow-up was measured at one, three, four, five, six, seven and eight years of follow-up.”

Also, per your comment regarding the subjective nature of self-reported highest weight since last follow-up, we agree that this is a limitation that warrants further consideration in the discussion section of our manuscript. We previously noted the limitations of using self-reported weight in our study at lines 282-287:

“To characterize the primary outcome self-reported weight was used, which may be prone to both intentional and unintentional misreporting (14, 15). Moreover, weight was measured only at baseline, while self-reported ‘highest weight since last follow-up’ was ascertained at all subsequent years of follow-up starting with year one. Accordingly, the self-report measure was used in conjunction with measured weight (at baseline) to compute percent change in weight from baseline. Nonetheless, we found measured weight at baseline to have acceptable correlation with highest reported weight in the time since last follow-up at year one.”

While we feel this text provides sufficient explanation of the limitation of using self-reported weight in our study, we would be happy to provide additional narrative at the behest of the reviewer.

**Comment 5:** Interacting and confounding: please discuss further other relevant unmeasured variables that are part of the causal mechanism proposed in your hypothesis: i.e. educational level, occupational status, psychosocial variables.

**Response to comment 5:** We further discuss other relevant unmeasured variables that may contribute to the disparity in postmenopausal obesity among NHBs and NHWs at lines 243-259 of the revised manuscript:

“Biological differences may include differences in energy expenditure and fatty acid metabolism that promote excess weight gain in NHBs relative to NHWs (2-4). Sociocultural differences between NHBs and NHWs may also contribute to the disparity in weight status. These include sociocultural differences in perceived weight status – it has been established that there are racial/ethnic differences in perceived weight status in NHB and NHW women. NHBs are more likely to perceive themselves as having a healthy body weight, even at higher BMIs, compared with NHWs (16), and this could contribute to higher rates of obesity among NHB women. Racial/ethnic differences in other sociocultural factors may also play a role. Black women are more likely than white women to experience lower socioeconomic positioning, thereby increasing their likelihood of weight gain throughout the lifespan (17). Other sociocultural differences in education level (5), annual household income (6), and physical activity level (7), have also been noted and may also contribute to the disparity in obesity prevalence among postmenopausal Black and White women, along with environmental factors. NHBs are more likely to reside in neighborhoods with limited access to healthy foods (11), and limited neighborhood walkability (12), both of which are associated with increased risk of weight gain. Lastly, it has been shown that differences in weight gain throughout the life-cycle may also contribute to racial/ethnic disparity in obesity among postmenopausal women. Black women experience greater weight gain than NHWs in early (18), middle (17, 19), and older adulthood (20), thereby placing them at greater risk of obesity than their White counterparts at virtually every age.”

**Comment 6:** Most respondents >90% were non-Hispanic White, how culd this affect your findings?

**Response to comment 6:** This was an important consideration for using data from the Women’s Health Initiative Observational Study. One of the reasons we chose this cohort was because they have such ample sample size. Thus, even with over 90% of the sample being non-Hispanic White, there were still nearly 7,500 non-Hispanic Black women with complete data in our study. This makes ours one of the largest prospective studies of weight gain in non-Hispanic Black women. Were the sample of non-Hispanic Blacks in our study smaller, we may have had concerns over sparse sample bias, particularly in our adjusted models. However, because of the large number of respondents in the WHI OS (n=93,676), it was possible to obtain a large sample of non-Hispanic Blacks, thereby minimizing the potential for sparse sample bias.

**Comment 7:** Discussion first sentence indicates that findings are similar to other studies, please clarigy the originality of this study.

**Response to comment 7:** Indeed, we noted that our overall finding is consistent with findings from other works in the first sentence of the discussion. However, we go on to note (at lines 216-219)

“the current study is unique in suggesting that differences in baseline weight status do *not* underlie racial/ethnic differences in the risk of postmenopausal weight gain. Rather, greater overall risk of weight gain in NHBs versus NHWs was largely attributable to a markedly higher risk of weight gain in NHBs with normal weight compared with NHWs with normal weight.”

**Comment 8:** Discussion: again, there is a rather poor explanation of potential causal mechanisms for the link between menopause, race and weight gain. This is crucial and needs further development.

**Response to comment 8:** Thank you for this insightful feedback. We agree that the discussion needed some improvement and we are grateful to you for providing some direction. At lines 243-259 of the revised manuscript, we expand upon our explanation of the potential causal mechanism for the link between menopause, race and weight gain:

“Biological differences may include differences in energy expenditure and fatty acid metabolism that promote excess weight gain in NHBs relative to NHWs (2-4). Sociocultural differences between NHBs and NHWs may also contribute to the disparity in weight status. These include sociocultural differences in perceived weight status – it has been established that there are racial/ethnic differences in perceived weight status in NHB and NHW women. NHBs are more likely to perceive themselves as having a healthy body weight, even at higher BMIs, compared with NHWs (16), and this could contribute to higher rates of obesity among NHB women. Racial/ethnic differences in other sociocultural factors may also play a role. Black women are more likely than white women to experience lower socioeconomic positioning, thereby increasing their likelihood of weight gain throughout the lifespan (17). Other sociocultural differences in education level (5), annual household income (6), and physical activity level (7), have also been noted and may also contribute to the disparity in obesity prevalence among postmenopausal Black and White women, along with environmental factors. NHBs are more likely to reside in neighborhoods with limited access to healthy foods (11), and limited neighborhood walkability (12), both of which are associated with increased risk of weight gain. Lastly, it has been shown that differences in weight gain throughout the life-cycle may also contribute to racial/ethnic disparity in obesity among postmenopausal women. Black women experience greater weight gain than NHWs in early (18), middle (17, 19), and older adulthood (20), thereby placing them at greater risk of obesity than their White counterparts at virtually every age.”

**Comment 9:** Discussion could be improved by adding and deepening the following angles: (i)deeper racial/ethnic sociocultural understandings, (ii) life-course perspective, (iii) gender perspective, (iv) social inequalities in health perspective.

**Response to comment 9:** Thank you for the opportunity to improve the discussion section by focusing on the areas you noted above. We have significantly revised the discussion of our manuscript. These changes are reflected at lines 243-267:

“Biological differences may include differences in energy expenditure and fatty acid metabolism that promote excess weight gain in NHBs relative to NHWs (2-4). Sociocultural differences between NHBs and NHWs may also contribute to the disparity in weight status. These include sociocultural differences in perceived weight status – it has been established that there are racial/ethnic differences in perceived weight status in NHB and NHW women. NHBs are more likely to perceive themselves as having a healthy body weight, even at higher BMIs, compared with NHWs (16), and this could contribute to higher rates of obesity among NHB women. Racial/ethnic differences in other sociocultural factors may also play a role. Black women are more likely than white women to experience lower socioeconomic positioning, thereby increasing their likelihood of weight gain throughout the lifespan (17). Other sociocultural differences in education level (5), annual household income (6), and physical activity level (7), have also been noted and may also contribute to the disparity in obesity prevalence among postmenopausal Black and White women, along with environmental factors. NHBs are more likely to reside in neighborhoods with limited access to healthy foods (11), and limited neighborhood walkability (12), both of which are associated with increased risk of weight gain. Lastly, it has been shown that differences in weight gain throughout the life-cycle may also contribute to racial/ethnic disparity in obesity among postmenopausal women. Black women experience greater weight gain than NHWs in early (18), middle (17, 19), and older adulthood (20), thereby placing them at greater risk of obesity than their White counterparts at virtually every age. This is consistent with the findings of our study. We found that postmenopausal NHBs had markedly higher rates of class I obesity, and more than twice the rates of class II and class III obesity, as NHWs. Recent evidence from the US nationally representative National Health and Nutrition Examination Survey (NHANES), which showed that in middle-(40-59 y) and older-(60+ y) aged women, the prevalence of obesity was an average of 20 percentage points higher in NHBs compared to NHWs (21), also supports this result. Taken together, these findings suggest that much of the divergence in the prevalence of obesity in NHW and NHB postmenopausal women occurs prior to age 40. Accordingly, efforts to reduce racial/ethnic disparities in obesity will require a focus on preventing excess weight gain in NHB women at earlier life stages, and particularly in those younger than age 40.”

**References**

1.	Reynolds RF, Obermeyer CM. Age at natural menopause in Spain and the United States: Results from the DAMES project. American Journal of Human Biology. 2005;17(3):331-40.

2.	Luke A, Dugas L, Kramer H. Ethnicity, energy expenditure and obesity: are the observed black/white differences meaningful? Current Opinion in Endocrinology, Diabetes and Obesity. 2007;14(5):370-3.

3.	Bower JF, Davis JM, Hao E, Barakat HA. Differences in transport of fatty acids and expression of fatty acid transporting proteins in adipose tissue of obese black and white women. American Journal of Physiology-Endocrinology and Metabolism. 2006;290(1):E87-E91.

4.	Barakat H, Davis J, Lang D, Mustafa SJ, McConnaughey MM. Differences in the Expression of the Adenosine A1 Receptor in Adipose Tissue of Obese Black and White Women. The Journal of Clinical Endocrinology & Metabolism. 2006;91(5):1882-6.

5.	Burke GL, Bild DE, Hilner JE, Folsom AR, Wagenknecht LE, Sidney S. Differences in weight gain in relation to race, gender, age and education in young adults: The CARDIA study. Ethnicity & Health. 1996;1(4):327-35.

6.	Ball K, Crawford D. Socioeconomic status and weight change in adults: a review. Social science & medicine. 2005;60(9):1987-2010.

7.	Jakicic JM, Powell KE, Campbell WW, Dipietro L, Pate RR, Pescatello LS, et al. Physical activity and the prevention of weight gain in adults: a systematic review. Medicine & Science in Sports & Exercise. 2019;51(6):1262-9.

8.	Ryan CL, Bauman K. Educational attainment in the United States: 2015. 2016.

9.	Semega JL, Fontenot KR, Kollar MA. Income and poverty in the United States: 2016. Current Population Reports. 2017(P60-259).

10.	Keadle SK, McKinnon R, Graubard BI, Troiano RP. Prevalence and trends in physical activity among older adults in the United States: a comparison across three national surveys. Preventive medicine. 2016;89:37-43.

11.	Walker RE, Keane CR, Burke JG. Disparities and access to healthy food in the United States: A review of food deserts literature. Health & place. 2010;16(5):876-84.

12.	Lim S, Harris TG. Neighborhood contributions to racial and ethnic disparities in obesity among New York City adults. Am J Public Health. 2015;105(1):159-65.

13.	Muscaritoli M, Anker S, Argiles J, Aversa Z, Bauer J, Biolo G, et al. Consensus definition of sarcopenia, cachexia and pre-cachexia: joint document elaborated by Special Interest Groups (SIG)“cachexia-anorexia in chronic wasting diseases” and “nutrition in geriatrics”. Clinical nutrition. 2010;29(2):154-9.

14.	Palta M, Prineas RJ, Berman R, Hannan P. Comparison of self-reported and measured height and weight. American journal of epidemiology. 1982;115(2):223-30.

15.	Rowland ML. Self-reported weight and height. The American journal of clinical nutrition. 1990;52(6):1125-33.

16.	Bennett GG, Wolin KY. Satisfied or unaware? Racial differences in perceived weight status. International Journal of Behavioral Nutrition and Physical Activity. 2006;3(1):40.

17.	Baltrus PT, Lynch JW, Everson-Rose S, Raghunathan TE, Kaplan GA. Race/ethnicity, life-course socioeconomic position, and body weight trajectories over 34 years: the Alameda County Study. Am J Public Health. 2005;95(9):1595-601.

18.	Lewis CE, Smith DE, Wallace DD, Williams OD, Bild DE, Jacobs Jr DR. Seven-year trends in body weight and associations with lifestyle and behavioral characteristics in black and white young adults: the CARDIA study. Am J Public Health. 1997;87(4):635-42.

19.	Insaf TZ, Shaw BA, Yucel RM, Chasan-Taber L, Strogatz DS. Lifecourse Socioeconomic Position and Racial Disparities in BMI Trajectories Among Black and White Women: Exploring Cohort Effects in the Americans’ Changing Lives Study. Journal of racial and ethnic health disparities. 2014;1(4):309-18.

20.	He XZ, Meng H. Changes in weight among US adults aged 70 and over, 1993 to 2002. Preventive medicine. 2008;47(5):489-93.

21.	Ogden CL, Carroll MD, Kit BK, Flegal KM. Prevalence of childhood and adult obesity in the United States, 2011-2012. Jama. 2014;311(8):806-14.
