# Supplementary material for: On the joint role of non-Hispanic Black race/ethnicity and weight status in predicting postmenopausal weight gain
Source: PLoS One. 2021 Mar 1;16(3):e0247821. doi: 10.1371/journal.pone.0247821 (PMC7920337; doi:10.1371/journal.pone.0247821)
Supplement: Attachment — Submitted filename: Response to Reviewers.docx [file pone.0247821.s007.docx]

Comments from Reviewer #1

**Comment 1:** Page 8, Lines 197 -199. The result for normal weight NHB, which was the strongest association of all the weight categories, was not described in the text. Also, the HR reported for class III obesity should be 1.39 (1.14,1.71) not 0.74 (0.59,0.92).

**Response to comment 1:** Thank you for bringing this to our attention. We have revised the text at lines 144-147 of the revised manuscript, which now reads:

“NHBs who were normal weight (HR: 1.78; 95% CI: 1.61, 1.96), overweight (HR: 1.49; 95% CI: 1.36, 1.63), class I obesity (HR: 1.47; 95% CI: 1.31, 1.64), class II obesity (HR: 1.73; 95% CI: 1.46, 2.04) and class III obesity (HR: 1.43; 95% CI: 1.17, 1.74) were more likely to gain weight than normal weight NHWs.”

All estimates were updated to reflect the results of carrying out the analyses with imputed data.

**Comment 2:** In this same paragraph, lines 192 to 205, the authors should mention the direction of the trends when they report the p-values. It appears to be a direct trend for the overall group and the NHW, but an inverse trend for the NHB.

**Response to comment 2:** Thank you for this suggestion. We remarked on the overall trend in unadjusted models at lines 133-135:

“The overall trend for a linear term for baseline weight status was significant (p<0.001), but the directionality was not consistent across categories of baseline weight status.”

We remarked on the overall trend in adjusted models at lines 138-139:

The was evidence of an inverse trend, which was significant (p=0.001).

We remarked on the trend in NHWs in unadjusted models at lines 142-143:

“…the trend was not significant (p=0.063).

We remarked on the trend in NHWs in adjusted models at lines 144-145:

The was evidence of an inverse trend, which was significant (p<0.001).

We remarked on the trend in NHBs in unadjusted models at lines 149-150:

“In unadjusted models, the trend was not significant in NHBs (p=0.060).”

We remarked on the trend in NHBs in adjusted models at lines 153-154:

There was evidence of an inverse trend, which was significant (p=0.004).

Please note that the results section was re-structured to address comment #4 below.

**Comment 3:** There are some numbers in the table that seem to have been included in error -- 01 after the HR and CI for NHB normal weight, 10 after the HR and CI for NHW overweight and 11 after the HR and CI for NHB overweight.

**Response to comment 3:** Thank you for catching this. We have double checked the values and removed the numbers that were included in error.

**Comment 4:** Page 9, paragraph starting with line 225. I'm confused about this paragraph. These data are supposed to be in Table 2, but I don't see any of these numbers in this table.

**Response to comment 4:** To address your comment, we reorganized our discussion of the findings presented in Table 2. In the first paragraph, we described the results of overall models. In the second paragraph, we describe the results of models using normal weight NHWs as the common referent group. In the third paragraph, we present the results of models comparing risk of weight gain in NHBs by category of baseline weight status to that of NHBs who were normal weight at baseline. Values have been updated to reflect the results of models run with imputed data. The updated presentation of results from Table 2 is given at lines 131-164.

**Comment 5:** Page 7, line 183. The sentence begins with "for the" rather than "The".

**Response to comment 5:** Thank you. We have made the correction.

Comments from Reviewer #2

**Comment 1:** I wonder if any respondents with disease, such as cancer, were also excluded from the sample for analysis. This is a serious condition that might affect weight gain/loss over time.
2).

**Response to comment 1:** We originally excluded individuals with underweight at baseline, those who identified as diabetic. In making our revisions, we have now excluded those with a history of cancer at baseline. This is described at lines 43-45.

**Comment 2:** It is reported that 965 cases are missing in BMI so excluded from the sample. However, there are a couple of statistical methods that can handle missing data, such as multiple imputations (MI) and full information maximum likelihood (FIML). In my opinion, it is necessary to compare the results with and without missing data and report the one without missing data.

**Response to comment 2:** Per your suggestion, we used multiple imputation with chained equations to impute missing data for BMI. We then retained these individuals in the analysis. All results were updated to reflect the results of analyses carried out using imputed data.

**Comment 3:** No information was provided for missing data of the variables in Table 1. This is crucial since it would affect sample size for each model in Table 2 and Appendix Table 1. Some variables seem to have missing data, such as household income. For example, numbers for household income of NHW does not add up to 76,208 (which is not equal to 70,909=9,622+31,183+21,744+8,360). Please report the total sample size for each variable in Table 1. Ideally, this can be handled properly by MI or FIML.

**Response to comment 3:** We have revised Table 1 to include the number missing for each variable.

**Comment 4:** Please report the sample size for each analysis in Table 2 and Appendix Table 1.

**Response to comment 4:** We have included in the titles of Table 2 and Appendix Table 1 their sample sizes.

**Comment 5:** Since additional analysis (Appendix Table 1) considered both race/ethnicity and age groups, please provide the sample sizes for age groups in Table 1.

**Response to comment 5:** We have provided sample sizes for each age group in Table 1.

**Comment 6:** Since the average ages reported in Table 1 are over 62, this might result in very small sample size for some categories, particularly for younger age groups for NHB, which might lead to low statistical power for some categories. This might be the reason of several nonsignificant results for NHB in Appendix Table 1.

**Response to comment 6:** We have examined the distribution of age in our sample and agree that an alternate categorization of age is warranted. We have therefore created four categories of age: 1) 49-54 (n=9,458); 2) 55-59 (n=13,402); 3) 60-64 (n=15,633); and 4) 65 and older (n=32,257).

**Comment 7:** Two exercise variables in the final adjusted model are likely to be highly correlated with each other. For that reason, it would be better if a global scale can be controlled for, instead of individual scales. Same concern over caloric intake at baseline and year 3 follow-up.

**Response to comment 7:** Thank you for your suggestion. We examined the correlation between the different physical activity intensities and found it to be poor. For example, the correlation between mild and moderate exercise was 0.13; the correlation between mild exercise and hard exercise was 0.05; and the correlation between moderate exercise and hard exercise was 0.18. In the absence of specific guidance for how to combine these measures, it seems prudent to maintain them as separate variables. Total energy intake at baseline and year three were moderately correlated (0.59), and therefore we only used baseline caloric intake in our adjusted models.

**Comment 8:** On page 7, it says that final adjusted models include several variables. However, in Table 1, there are additional variables not mentioned, such as waist circumference and education. This might confuse readers. If they were not used in the final models, it would be better not to be included in Table 1 for descriptive statistics.

**Response to comment 8:** We have now included in our fully-adjusted models all of the variables included in Table 1. Age is included as a continuous variable. We no longer include in our adjusted models variables that are not presented in Table 1.

**Comment 9:** Waist circumference can also be used to assess obesity. In fact, it might be more meaningful than BMI: <https://www.hsph.harvard.edu/obesity-prevention-source/obesity-definition/abdominal-obesity/>. If it was measured more than one time, like weight and height, then it would be extremely meaningful if both BMI and waist circumference can be analyzed and compared across race/ethnicity.

**Response to comment 9:** Waist circumference was measured only at baseline and year 3. Without more time points available for waist circumference, it would not be possible to repeat our analyses using waist circumference.

**Comment 10:** Menopause, on average, is likely to happen at early 50s so linking menopause with weight gain among women in this data seems to be a very ambitious stretch. Because of this reason, it is crucial to carefully examine the patterns of age 50-54 for both NHW and NHB, but there is a concern over sample size particularly for this age group of NHB. As a matter of fact, there are few significant results once adjusted for several factors especially when NHB was compared with NHW in the same weight status. I think the introduction needs to be revised so that it can actually lead to the main hypotheses for this study.

**Response to comment 10:** Per your suggestion we have significantly revised the introduction section to better support the objectives of this study.

**Comment 11:** In Appendix Table 1, readers need to compare the numbers horizontally and vertically for NHB but it is very confusing. Please consider summarizing numbers in a separate table for the comparison between NHB and NHW or reorganize Appendix Table 1 for better readability.

**Response to comment 11:** We have split Appendix Table 1 into two tables: Appendix Table 1 and Appendix Table 2. The results of models comparing NHBs to NHWs are presented in Appendix Table 1. The results of overall models, and those from stratum-specific models in NHBs are presented in Appendix Table 2.

**Comment 12:** Please do not use a subjective term, such as “just” on page 3 line 75.

**Response to comment 12:**  We have removed “just”.

**Comment 13:** In table 2, it seems that there are some numbers that might not belong to the table. Check the numbers positioned next to 95% C.I. for NHW and NHB under the adjusted models. Please review the numbers in tables.

**Response to comment 13:** Thank you for catching this. We have removed the erroneous numbers from Table 2.
