# Supplementary material for: On the joint role of non-Hispanic Black race/ethnicity and weight status in predicting postmenopausal weight gain
Source: PLoS One. 2021 Mar 1;16(3):e0247821. doi: 10.1371/journal.pone.0247821 (PMC7920337; doi:10.1371/journal.pone.0247821)
Supplement: Attachment — Submitted filename: Response to Reviewer Comments.docx [file pone.0247821.s008.docx]

*Miscellaneous comments*

**Comments to the Author**

Please reference Appendix Tables 3 and 4 in the text

**Response to comment:** Appendix Table 4 is referenced at line 253. Appendix Table 3 is referenced at line 80.

1. If the authors have adequately addressed your comments raised in a previous round of review and you feel that this manuscript is now acceptable for publication, you may indicate that here to bypass the “Comments to the Author” section, enter your conflict of interest statement in the “Confidential to Editor” section, and submit your "Accept" recommendation.

Reviewer #1: (No Response)

Reviewer #2: All comments have been addressed

**Response to comment:** We have addressed each of the additional concerns raised by the two reviewers.

2. Is the manuscript technically sound, and do the data support the conclusions?

The manuscript must describe a technically sound piece of scientific research with data that supports the conclusions. Experiments must have been conducted rigorously, with appropriate controls, replication, and sample sizes. The conclusions must be drawn appropriately based on the data presented.

Reviewer #1: Partly

Reviewer #2: Yes

**Response to comment:** We believe that our revised manuscript is technically sound and that the data support the conclusions.

3. Has the statistical analysis been performed appropriately and rigorously?

Reviewer #1: I Don't Know

Reviewer #2: Yes

**Response to comment:** We have added extensive text to better explain our statistical approach.

4. Have the authors made all data underlying the findings in their manuscript fully available?

The [PLOS Data policy](https://urldefense.com/v3/__http:/www.plosone.org/static/policies.action*sharing__;Iw!!OlavHw!v92q9ElcJhI8ekjNCeiTiaoUO-v4d6OveeeNxTUZEEc7lGJJDavQtZefbbIukMC8_06zZHQ$) requires authors to make all data underlying the findings described in their manuscript fully available without restriction, with rare exception (please refer to the Data Availability Statement in the manuscript PDF file). The data should be provided as part of the manuscript or its supporting information, or deposited to a public repository. For example, in addition to summary statistics, the data points behind means, medians and variance measures should be available. If there are restrictions on publicly sharing data—e.g. participant privacy or use of data from a third party—those must be specified.

Reviewer #1: Yes

Reviewer #2: Yes

**Response to comment:** Thank you for approving this portion of the manuscript.

5. Is the manuscript presented in an intelligible fashion and written in standard English?

PLOS ONE does not copyedit accepted manuscripts, so the language in submitted articles must be clear, correct, and unambiguous. Any typographical or grammatical errors should be corrected at revision, so please note any specific errors here.

Reviewer #1: Yes

Reviewer #2: Yes

**Response to comment:** Thank you for approving this portion of the manuscript.

*Specific Comments from Reviewer #1*

**Comment 1:** In re-reviewing this manuscript, I realized that the definition of the primary outcome raised a significant concern in the interpretation of the results, specifically the results stratified by baseline weight. The outcome is defined as > 10% increase in weight from baseline, which means that, for example, a woman who started out at a weight of 120 lbs (normal BMI), would meet the threshold for the outcome if she gained 13 pounds, whereas a woman who had a baseline weight of 250 lbs (obese class III) would have to gain at least 25 pounds. To conclude that “the risk of weight gain was lower in women with obesity at baseline than those with normal weight” may not be accurate. It is possible that the very obese women gained on average 20 lbs but many of them did not meet the 10% increase threshold while the normal weight women could have had an average weight gain of 15 lbs, with more of them meeting the 10% threshold.

At a minimum, the authors should revise the manuscript to describe the outcomes more precisely, i.e., in the text they should consistently describe the outcome as “risk of > 10% weight gain” rather than simply “risk of weight gain”. The discussion should also acknowledge the limitations of defining the outcome as a 10% increase in weight rather than an absolute change in weight.

Preferably, the authors should re-analyze the data with the outcome as a continuous variable, looking at the absolute change in weight and determining if the conclusions are similar to those reached when defining the outcome as a weight gain of at least 10% change from baseline.

**Response to comment 1:** Thank you for the opportunity to improve our work. We used a relative measure of weight gain so that the threshold for weight gain would be similar across categories of baseline BMI. This approach has been used previously in the Women’s Health Initiative Observational Study [1]. Moreover, it is a foil to the criteria for significant weight-loss (10% or more) as described by Wing and Hill [2]. We have included as a limitation that a relative measure of weight gain was used, which meant that women who were heavier at baseline would need to gain more weight in order to achieve a 10% or more weight gain. This clarifying language is given at lines 246-252:

“Lastly, it is a limitation of this study that the outcome was defined as a relative measure (≥10%). This definition of significant weight gain corresponds to the widely-used definition of significant weight loss (≥10%) as defined by Wing and Hill of the National Weight Loss Consortium [2]. Use of a relative measure of weight loss meant that women who were heavier at baseline would need to gain more weight than those who weighed less in order to achieve a 10% weight gain. Nonetheless, in sensitivity analysis using ≥10 pound weight loss as the primary outcome, the results were nearly identical to those presented from the primary analysis.”

In addition, we carried out sensitivity analyses using ≥10 pounds as the primary outcome. Results of these sensitivity analyses are presented in Appendix Table 4. As shown, results from these models were very similar to those presented in Table 2.

**Comment 2:** Introduction, lines 24-27: This is a run-on sentence and its meaning is not entirely clear.

**Response to comment 2:** We revised the run-on sentence at lines 25-28. Thank you for bringing this to our attention.

**Comment 3:** Approach, line 75: Delete the word “in”.

**Response to comment 3:** Done.

**Comment 4:** Results, line 129: As described above, the results should be described more precisely “…were more likely to have a weight gain of > 10%” here and throughout the paper.

**Response to comment 4:** Thank you. We have made the suggested correction throughout the revised manuscript.

**Comment 5:** Results, line 141-162: Many of the numbers in these two paragraphs do not exactly match the numbers in Table 2. All numbers should be checked for accuracy.

**Response to comment 5:** We have double checked all reported values and revised where needed.

**Comment 6:** Results, line 151: NHW in this line should be NHB.

**Response to comment 6:** Thank you for catching this typographical error. We have made the correction at line 155 of the revised manuscript.

**Comment 7:** Results, line 157: It is not clear what is meant when the authors state “HRs in unadjusted models were similar..” Similar to what?

**Response to comment 7:** Thank you for the chance to clarify. Results were similar in crude and adjusted models. We have noted this at lines 163-164:

“HRs in unadjusted and adjusted models were similar, but there was no significant trend in unadjusted models.”

*Specific Comments from Reviewer #2*

**Comment 1:** Reviewer #2: 1) Please provide the reason for the choice of 10 data sets, not 20 or 5, for multiple imputations briefly in text. Also, please report what statistical program was used for analysis.

Thank you for the opportunity to clarify our approach. We have noted at lines 76-79 that:

“The choice to impute 10 datasets was made as a conservative application of the approach used by Gao, Wilson and Hepgul et al., who reported imputing 20 copies of their data in their 2020 study published in the Journal of the American Medical Association [3].”

We have also noted at lines 113-114 that:

“All analyses were carried out in Stata (Version 16, Stata Corp, College Station, Texas, USA).”

**Comment 2:** Please briefly discuss the differences in the results with and without multiple imputations. It is important to know if there are substantially different estimations between two approaches (listwise vs. MI).

**Response to comment 2:** The imputed results were similar to those obtained when the data were not imputed. There were no major differences in the qualitative interpretation of the findings. We repeated the analyses shown in Table 2 using a complete-case analysis. These findings are presented in Appendix Table 3. As shown, the point estimates and confidence intervals are very similar to those presented in Table 2.

**Comment 3:** In Table 2 and Appendix Table 1, the reference group is always NHW with normal weight. Please provide the reasoning for the choice. Why not compare NHB with NHW within each weight status group? It would be more interesting, for example, if NHB with overweight was compared to NHB with overweight, not to NHW with normal weight.

**Response to comment 3:** Having normal weight NHWs as the reference group allows us to determine the joint effect of NHB race/ethnicity and baseline weight status. In our stratum-specific models, NHBs with normal weight at baseline are the reference group, and thus, we have compared NHB overweight to NHB normal weight. Respectfully, your suggestion to compare NHB with overweight to NHB with overweight doesn’t make sense.

**Comment 4:** 4) Please provide the reasoning for 10% or more increase in weight as the outcome. The study is assuming that 10% or more weight gain for someone with normal weight at baseline has the same effect for someone with overweight or obesity at baseline. But gaining 10% or more weight would be much more difficult and harmful for someone with obesity than for someone with normal weight.

We are happy to note at lines 246-252 that:

“Lastly, it is a limitation of this study that the outcome was defined as a relative measure (≥10%). This definition of significant weight gain corresponds to the widely-used definition of significant weight loss (≥10%) as defined by Wing and Hill of the National Weight Loss Consortium [2]. Use of a relative measure of weight loss meant that women who were heavier at baseline would need to gain more weight than those who weighed less in order to achieve a 10% weight gain. Nonetheless, in sensitivity analysis using ≥10 pound weight gain as the primary outcome, the results were similar to those presented from the primary analysis.”

We present the results of these sensitivity analyses in Appendix Table 4.

**References**

[1] Ford C, Chang S, Vitolins MZ, Fenton JI, Howard BV, Rhee JJ, et al. Evaluation of diet pattern and weight gain in postmenopausal women enrolled in the Women’s Health Initiative Observational Study. British journal of nutrition. 2017;117:1189-97.

[2] Wing RR, Phelan S. Long-term weight loss maintenance–. The American journal of clinical nutrition. 2005;82:222S-5S.

[3] Gao W, Wilson R, Hepgul N, Yi D, Evans C, Bajwah S, et al. Effect of Short-term Integrated Palliative Care on Patient-Reported Outcomes Among Patients Severely Affected With Long-term Neurological Conditions: A Randomized Clinical Trial. JAMA Network Open. 2020;3:e2015061-e.
